# Comparison of six risk scores for stroke-associated pneumonia in patients with acute ischemic stroke: A systematic review and Bayesian network meta-analysis

**DOI:** 10.3389/fmed.2022.964616

**Published:** 2022-10-12

**Authors:** Xuemin Zhang, Lu Xiao, Liqing Niu, Yongchao Tian, Kuang Chen

**Affiliations:** ^1^First Teaching Hospital of Tianjin University of Traditional Chinese Medicine, Tianjin, China; ^2^National Clinical Research Center for Chinese Medicine Acupuncture and Moxibustion, Tianjin, China

**Keywords:** risk prediction, risk score, ischemic stroke (IS), network meta-analysis, stroke-associated pneumonia (SAP)

## Abstract

**Background:**

Stroke-associated pneumonia (SAP) is one of the major causes of death after suffering a stroke. Several scoring systems have been developed for the early prediction of SAP. However, it is unclear which scoring system is more suitable as a risk prediction tool. We performed this Bayesian network meta-analysis to compare the prediction accuracy of these scoring systems.

**Methods:**

Seven databases were searched from their inception up to April 8, 2022. The risk of bias assessment of included study was evaluated by the QUADAS-C tool. Then, a Bayesian network meta-analysis (NMA) was performed by R 4.1.3 and STATA 17.0 software. The surface under the cumulative ranking curve (SUCRA) probability values were applied to rank the examined scoring systems.

**Results:**

A total of 20 cohort studies involving 42,236 participants were included in this analysis. The results of the NMA showed that AIS-APS had excellent performance in prediction accuracy for SAP than Chumbler (MD = 0.030, 95%CI: 0.004, 0.054), A2DS2 (MD = 0.041, 95% CI: 0.023, 0.059), ISAN (MD = 0.045, 95% CI: 0.022, 0.069), Kwon (MD = 0.077, 95% CI: 0.055, 0.099) and PANTHERIS (MD = 0.082, 95% CI: 0.049, 0.114). Based on SUCRA values, AIS-APS (SUCRA: 99.8%) ranked the highest.

**Conclusion:**

In conclusion, the study found that the AIS-APS is a validated clinical tool for predicting SAP after the onset of acute ischemic stroke.

**Systematic review registration:**

https://www.crd.york.ac.uk/PROSPERO/display_record.php?RecordID=292375, identifier: CRD42021292375.

## Introduction

Stroke-associated pneumonia (SAP) which was introduced for the first time by Hilker et al. (1) in 2003 is one of the major causes of death after suffering a stroke. The morbidity of SAP varies from 8.6% (2) to 21.4% (3). It significantly increased the mortality, length of hospitalization, and economic burden among these patients (4–7). Therefore, early identification of SAP high-risk groups and timely therapy are crucial.

As various approaches (8, 9) were well-recognized in research and clinical practice which may lead to delayed or inappropriate antibiotic therapy, Pneumonia in Stroke Consensus [PISCES] Group defined SAP as the spectrum of lower respiratory tract infections within the first 7 days after stroke onset in 2015 (10). Currently, the modified criteria of the Centers for Disease Control and Prevention (mCDC criteria) (11) was widely used for the diagnosis of SAP (12, 13). Meanwhile, various risk factors for SAP have been reported in recent years, which include mechanical ventilation, atrial fibrillation, pre-existing respiratory disease, smoking, pre-existing heart disease, stroke severity, stroke-induced immunodepression and dysphasia etc. (14, 15). Combined with the SAP risk factors, several scoring systems such as A2DS2 (Age, Atrial fibrillation, Dysphagia, Sex, Stroke Severity) in Germany (16), ISAN (Prestroke Independence, Sex, Age, National Institutes of Health Stroke Scale) in UK (4), AIS-APS (Acute Ischaemic Stroke-Associated Pneumonia Score) in China (17), Kwon (Pneumonia Score) in Korea (18), PANTHERIS (Preventive Antibacterial Therapy in Acute Ischaemic Stroke) in Germany (19), Chumbler (Veteran's Health Administration cohort score) in USA (20) have been developed for the early prediction. On the one hand, some original research papers have compared several of these scoring systems but got contradictory results. The ranking of these scoring systems varies. On the other hand, the head-to-head clinical trials comparing the accuracy of these scoring systems are lacking up to now. Only a few original research papers (21) compared all scoring systems. Two systematic reviews (22, 23) determined the predictive performance of A2DS2, ISAN and AIS-APS, but do not compare the predictive accuracy among these scoring systems. It is still unclear which scoring system is more suitable as a risk prediction tool for SAP.

The network meta-analysis allows a quantitative comparison of multiple interventions to select the best option. Therefore, we compared the six scoring systems for rational predicting the risk of SAP based on the Bayesian network meta-analysis method.

## Materials and methods

### Study registration

This study was prepared under the guidance of the Preferred Reporting Items for Systematic Review and Meta-Analysis (PRISMA) guidelines. The study was prospectively registered on the PROSPERO platform (https://www.crd.york.ac.uk/prospero/) with an assigned registration number CRD42021292375.

### Inclusion and exclusion criteria

#### Inclusion criteria

1) Retrospective or prospective cohort studies.
2) Age ≥15 years, ischemic stroke patient.
3) The reference standards for SAP diagnosis according to the clinical, laboratory, and radiological examinations according to the modified criteria of the Centers for Disease Control and Prevention.
4) All studies compared the prediction accuracy of two or more selected scoring systems (A2DS2, ISAN, AIS-APS, Kwon, PANTHERIS, and Chumbler) for patients with SAP.

#### Exclusion criteria

1) Transient ischemic attack (TIA) Patients.
2) Studies included special populations (oncology patients, pregnant women, patients using immunosuppressive drugs, liver cirrhosis, etc.).
3) Studies with incomplete research data, unable to extract valid data.
4) Studies with duplicate publications or duplicate data.

### Search strategy

A systematic and comprehensive search was performed using electronic databases of PubMed (MEDLINE), Embase, Web of Science, China National Knowledge Infrastructure (CNKI), China Science and Technology Journal Database (VIP), China Biology Medicine disc (CBM) and Wanfang data from their inception up to April 8th, 2022. In addition, relevant Meta-analysis and systematic review were manually retrieved to track references of included literatures. As for studies with incomplete data, we would contact the authors of the studies. If valid data was still not available, the study would be excluded.

### Literature selection and data extraction

Two investigators (XM Zhang and L Xiao) independently screened the papers by checking the titles, abstracts, and keywords. Then, full texts were read to select studies meeting eligibility criteria. Any inconsistencies during the entire study selection were resolved by thorough discussion or the third investigator (YC Tian). The information including eligible study characteristics (e.g., first author and year of publication), participant characteristics (e.g., gender, age, and sample), details of interventions (e.g., scoring systems), outcome data, and factors to evaluate risk of bias were extracted and entered into the spreadsheet.

### Risk of bias assessment

The risk of bias was evaluated using the QUADAS-C (24) risk of bias assessment tool (http://www.bristol.ac.uk/population-health-sciences/projects/quadas/quadas-c). QUADAS-C (C stands for comparative) is an extension to QUADAS-2 for assessing risk of bias in comparative accuracy studies. The QUADAS-C tool retains the same 4-domain structure of QUADAS-2 (Patient Selection, Index Test, Reference Standard, and Flow and Timing) and comprises additional questions to each QUADAS-2 domain. A risk-of-bias judgment for comparative accuracy requires a risk-of-bias judgment for the accuracy of each test (resulting from QUADAS-2) and additional criteria specific to test comparisons. Two investigators (XM Zhang and L Xiao) independently evaluated the risk of bias of the included studies and cross-checked the results.

### Statistical analysis

R software version 4.1.3 and STATA software were employed to compute calculations and prepare graphs. The gemtc packages and rjags packages were utilized to compute a Markov chain Monte Carlo (MCMC) simulation. Odds ratios (OR) with 95% confidence intervals (CIs) were used for dichotomous data. For continuous variables, mean differences (MD) with 95% CIs. Initially, both fixed effects and random effects models were fitted. Preset model parameters: four chains were used for simulation analysis, 20,000 annealing times, a step size of one, and 50,000 simulation iterations. The deviance information criterion (DIC) was used to judge the degree of model fit (25). A lower DIC score meant a better fit. After the subsequent analysis was performed. Consistency in the entire network was evaluated by calculating the unrelated mean effects (UME) model (26, 27). Consistency between direct and indirect comparison was analyzed by the node-splitting method (28), and *P* < 0.05 indicated a significant inconsistency for a specific comparison. The *I*^2^ statistic was used to assess the heterogeneity between studies, with a cut-off point of 50%. The network graph constructed by STATA software represented a comparative relationship between different interventions. The surface under the cumulative ranking curves (SUCRA) were calculated to present the ranking probability of different scoring systems. The range of SUCRA was from 0 to 100% (29, 30). After that, publication bias were reflected by funnel plots (31, 32).

## Results

**Figure 1:**
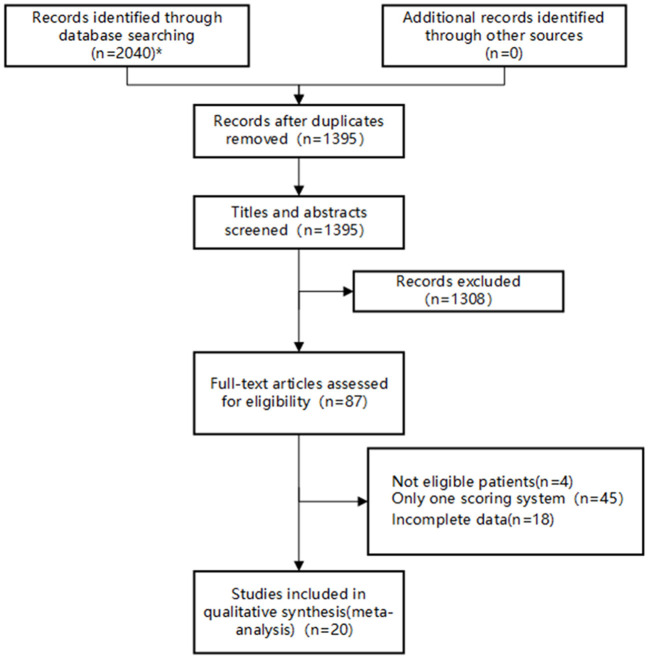
Flow diagram of study inclusion. *The databases searched and the number of studies retrieved are as follows: PubMed (*n* = 826), Embase (*n* = 48), Web of Science (*n* = 769), CNKI (*n* = 40), Wan Fang Data (*n* =70), VIP (*n* = 254), and SinoMed (*n* = 33).

A total of 2,040 studies were identified from the search at first. After removing duplicates, 1,395 remained. By screening titles and abstracts, 1,308 studies were excluded because they were reviews, irrelevant studies, and animal experiments. Afterwards, 87 relevant studies were reviewed for eligibility by full-text evaluations. Finally, 20 studies that met the inclusion criteria were included in our Bayesian NMA. 67 records were excluded for the following reasons: (1) intracerebral hemorrhage patients (*n* = 4); (2) The study examined only one of the selected scoring systems (*n* = 45); (3) incomplete data (*n* = 18). The literature selection process was illustrated in Figure 1.

### Study characteristics

**Figure 2:**
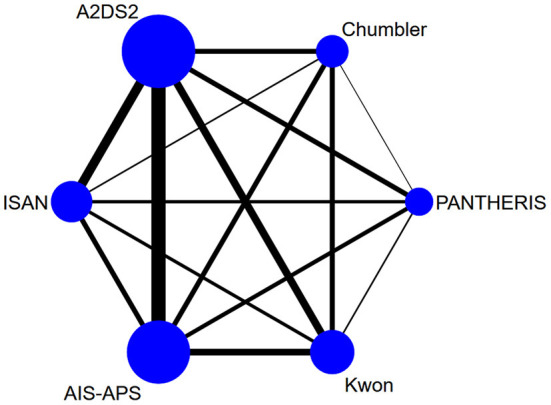
Network graph of the area under the receiver operating characteristic (AUC).

The Bayesian NMA was performed using 20 cohort studies with a total of 42,236 patients and their sample sizes varying from 40 to 14,400 participants. Twenty studies were conducted in China (13) (17, 21, 33–43), Singapore (1) (44), Spain (1) (45), Egypt (1) (13), Indonesia (1) (46), the United Arab Emirates (1) (47), the United Kingdom (1) (48), and France (1) (2) and published between 2013 and 2021. The study by Ji et al. (17) contained three validation cohorts, which were split to represent. The details of the study characteristics were depicted in Table 1. The network diagram is presented in Figure 2. Lines width is proportional to the number of references including the comparison. Dots area is proportional to the number of patients in the scoring systems.

**Table 1:** Characteristics of the studies included in this meta-analysis.

**Study**	**Country**	**Study** **design**	**Sample** **size**	**Study participants**	**Mean** **age (in** **years)**	**Male** **(%)**	**Pneumonia (*n*)**	**Reference** **standard** **assessment**	**Index test**
Cugy (2)	France	Retrospective	1,960	Age ≥15 years, acute ischemic stroke confirmed on brain CT or MRI	67.85	60.00%	168	Pneumonia diagnosed by the clinician team based on clinical (lung auscultation and percussion, presence of fever, purulent tracheal secretion), microbiological (tracheal specimens, blood cultures), and imaging findings	A2DS2, ISAN, Kwon
Elhasin et al. (47)	United Arab Emirates	Prospective	200	NA	NA	NA	42	NA	A2DS2, ISAN
Han (39)	China	Retrospective	95	Age >60 years, ischemic stroke diagnosed based on the Chinese guidelines for diagnosis and treatment of acute ischemic stroke 2014 criteria and confirmed by brain CT or MRI	73.15	54.74%	22	Differential diagnosis and treatment of respiratory system disease complications, Wu Xiaojun and Nie Hanxiang	A2DS2, AIS-APS
Hang (21)	China	Retrospective	1,427	Age ≥18 years, ischemic stroke onset ≤ 7 days	68.88	67.70%	395	Diagnosis according to modified criteria of the Centers for Disease Control and Prevention.	A2DS2, ISAN, AIS-APS, Kwon, PANTHERIS, Chumbler
Helmy et al. (13)	Egypt	Prospective	70	Age ≥18 years, acute ischemic stroke (an episode of neurological dysfunction caused by focal cerebral ischemic injury based on symptoms persisting ≥24 h	60	47.10%	26	Diagnosis according to modified criteria of the Centers for Disease Control and Prevention	A2DS2, AIS-APS, PANTHERIS
Hu (36)	China	Retrospective	246	ischemic stroke diagnosed based on the Diagnostic points for various types of cerebrovascular diseases (1995) criteria and confirmed by brain CT or MRI, onset ≤ 72 h	65.73	74.79%	52	Chinese expert consensus on the diagnosis and management of stroke-associated pneumonia 2010	A2DS2, AIS-APS, Kwon, Chumbler
Huang et al. (41)	China	Retrospective	340	Age ≥18 years, diagnosis of acute ischemicstroke (AIS) confirmed by CT or MRI within 24 h after admission, onset ≤ 24 h	66.4	65.60%	50	Diagnosis according to modified criteria of the Centers for Disease Control and Prevention	A2DS2, ISAN, PANTHERIS
Ji et al. (17) derivation cohort	China	Retrospective	8,850	Age ≥18 years, hospitalized with a primary diagnosis of AIS according to World Health Organization criteria, stroke confirmed by head CT or brain MRI, direct admission to hospital from physician clinic or emergency department	66	61.60%	1,007	SAP diagnosed according to the Centers for Disease Control and Prevention criteria for hospital acquired pneumonia on basis of clinical and laboratory indices of respiratory tract infection (fever, cough, auscultatory respiratory crackles, new purulent sputum, or positive sputum culture), supported by typical chest X-ray findings	A2DS2, AIS-APS, Kwon, Chumbler
Ji et al. (17) external validation cohort	China	Retrospective	3,037	Age ≥18 years, hospitalized with a primary diagnosis of AIS according to World Health Organization criteria, stroke confirmed by head CT or brain MRI, direct admission to hospital from physician clinic or emergency department				SAP diagnosed according to the Centers for Disease Control and Prevention criteria for hospital acquired pneumonia on basis of clinical and laboratory indices of respiratory tract infection (fever, cough, auscultatory respiratory crackles, new purulent sputum, or positive sputum culture), supported by typical chest X-ray findings	A2DS2, AIS-APS, Kwon, Chumbler
Ji et al. (17) internal validation cohort	China	Retrospective	5,882	Age ≥18 years, hospitalized with a primary diagnosis of AIS according to World Health Organization criteria, stroke confirmed by head CT or brain MRI, direct admission to hospital from physician clinic or emergency department	66	62.50%	662	SAP diagnosed according to the Centers for Disease Control and Prevention criteria for hospital acquired pneumonia on basis of clinical and laboratory indices of respiratory tract infection (fever, cough, auscultatory respiratory crackles, new purulent sputum, or positive sputum culture), supported by typical chest X-ray findings	A2DS2, AIS-APS, Kwon, Chumbler
Harms et al. (19)	China	Prospective	276	The diagnosis of cerebrovascular disease was based on clinical presentations combined with assessments of brain computed tomography (CT) or magnetic resonance imaging (MRI) by physicians.	70.35	63.77%	67	SAP was diagnosed according to the recommendations from the pneumonia in stroke consensus group (Diagnosis according to modified criteria of the Centers for Disease Control and Prevention)	A2DS2, ISAN, AIS-APS, PANTHERIS
Li (40)	China	Retrospective	3,104	Age ≥18 years, ischemic stroke onset ≤ 7 days	65	62.10%	100	Hospital acquired pneumonia	A2DS2, ISAN, AIS-APS, Kwon
Luo (37)	China	Retrospective	203	Ischemic stroke diagnosed based on the 4th National Conference on Cerebrovascular Diseases (1995) criteria and confirmed by brain CT or MRI, onset ≤ 7 days	65.37	64.04%	46	Diagnosis according to modified criteria of the Centers for Disease Control and Prevention.	A2DS2, AIS-APS
Ramírez-Moreno (45)	Spain	Prospective	285	Participants with acute ischemic stroke confirmed on MRI, onset ≤ 72 h	71.07	62.10%	45	SAP diagnosed on the basis of clinical and laboratory indices of respiratory tract infection, supported by typical chest X-ray findings	A2DS2, ISAN
Rehan et al. (48)	United Kingdom	Prospective	213	NA	NA	NA	NA	Development of probable or definite SAP was recorded according to CDC criteria.	A2DS2, ISAN
Shan (34)	China	Retrospective	252	Age ≥18 years, ischemic stroke diagnosed based on the Chinese guidelines for diagnosis and treatment of acute ischemic stroke 2014 criteria, onset ≤ 72 h	68.32	57.50%	47	Chinese expert consensus on the diagnosis and management of stroke-associated pneumonia 2010	A2DS2, AIS-APS, Kwon, PANTHERIS
Siregar et al. (46)	Indonesia	Retrospective	40	Participants with acute ischemic stroke confirmed on MRI	NA	NA	NA	Diagnosis according to modified criteria of the Centers for Disease Control and Prevention.	A2DS2, AIS-APS
Tu (44)	Singapore	Retrospective	731	Adult AIS patients, onset ≤ 4.5 h, Diagnosis had to be made by a neurologist and infarcts confirmed by neuroimaging.	NA	NA	40	The definition of stroke-associated pneumonia was based on the criteria by the Pneumonia in Stroke Consensus Group	A2DS2, ISAN, AIS-APS, Chumbler
Wang (33)	China	Retrospective	338	Ischemic stroke diagnosed based on the 4th National Conference on Cerebrovascular Diseases (1995) criteria and confirmed by brain CT or MRI, onset ≤ 72h	77	51.50%	125	Chinese expert consensus on the diagnosis and management of stroke-associated pneumonia 2010	A2DS2, ISAN, AIS-APS, PANTHERIS
Yang (38)	China	Retrospective	86	age≥18 years, ischemic stroke diagnosed based on 4th National Conference on Cerebrovascular Diseases (1995) criteria and confirmed by brain CT or MRI	61.3	55.80%	19	Hospital acquired pneumonia within 7 days	A2DS2, AIS-APS
Zhang et al. (43)	China	Retrospective	14,400	China National Stroke Registry	NA	62.00%	1,630	Diagnosis according to modified criteria of the Centers for Disease Control and Prevention.	A2DS2, ISAN, AIS-APS
Zhang (35)	China	Retrospective	201	Age ≥18 years, acute ischemic stroke (an episode of neurological dysfunction caused by focal cerebral ischemic injury based on symptoms persisting ≥24 h, confirmed by brain CT or MRI, onset ≤ 7 days	73.4	64.40%	31	Diagnosis according to modified criteria of the Centers for Disease Control and Prevention.	A2DS2, AIS-APS

### Quality assessments of studies

**Figure 3:**
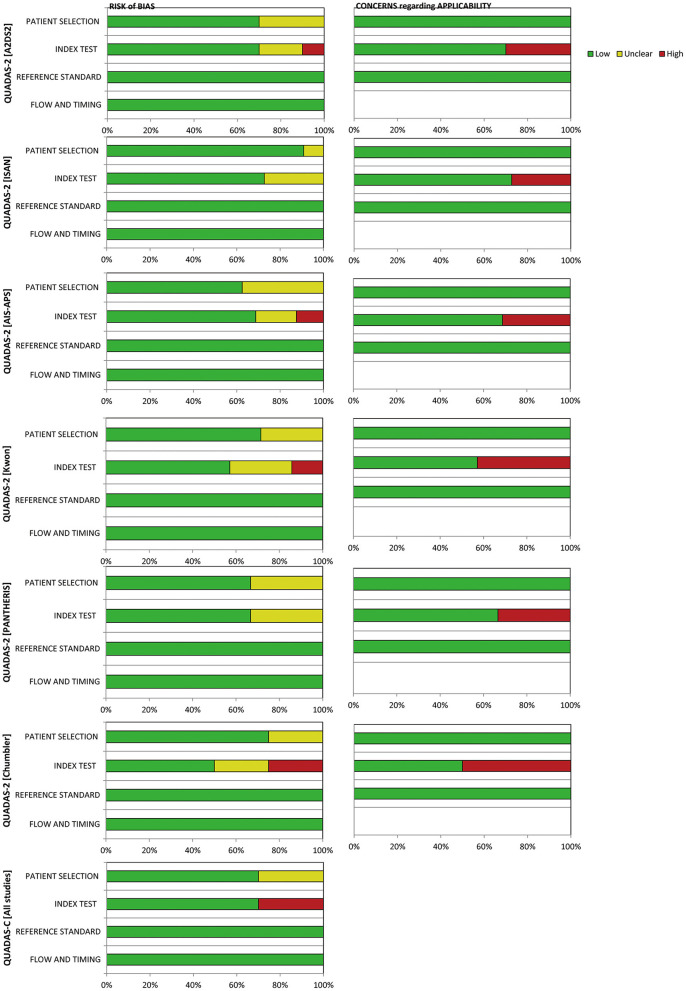
Risk-of-bias graph.

We used the QUADAS-C tools to conduct the quality evaluation. All of the studies were in full paired design (each patient receiving all of the index tests in the studies). Six studies (33, 34, 36–39) were assessed as “unclear risk” in terms of patient selection due to unreported whether the selection of patients was consecutive or not. Six studies (2, 21, 33, 36, 37, 39) were assessed as “high risk” in terms of index test due to unable to determine whether to interpret the index tests results without knowledge of the results of the reference standard. Further details of the risk of bias assessment are shown in Figure 3.

### Model selection

**Figure 4:**
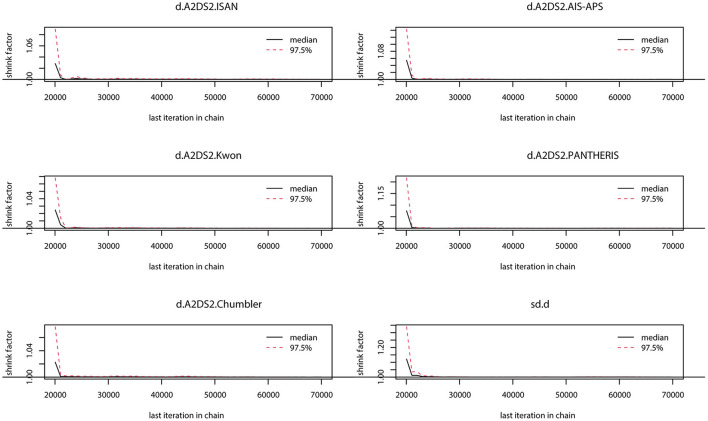
Convergence diagnosis graph. The value of the potential scale reduction factor (PSRF) tends to 1 indicating a satisfactory degree of model convergence.

**Figure 5:**
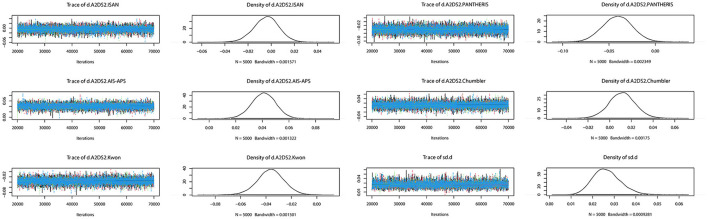
Trajectory and density graph. Each MCMC chain in the trajectory diagram has reached stable fusion from the beginning part, and the overlapping area accounts for most of the chain fluctuation range in the subsequent calculation, and the fluctuation of a single chain cannot be recognized by the naked eye, so the model convergence degree is satisfactory. The distribution of the graphs in the density map is normal, and the Bandwidth value tends to 0 and reaches stability, and the model convergence degree is satisfactory when combined with the results of the trajectory map.

The preliminary model fit showed fixed effects model DIC= 165.8, *I*^2^ = 50% and random effects model DIC = 129.5, *I*^2^ = 12%, so a random effects model was used for the network meta-analysis. The convergence diagnosis graph and trajectory and density graph of the random effects model were shown in Figures 4, 5.

### Inconsistency test

The consistency in the entire network was evaluated by calculating the UME model, the results showed DIC= 129.5. The local inconsistency test used the node-splitting method, and all the results showed *P* > 0.05 (Table 2). Overall, no inconsistency was found. The results of NMA are reliable.

**Table 2:** The results of node-splitting method for inconsistency test.

**Comparison**	**Direct**	**Indirect**	***P***
AIS-APS vs. ISAN	0.032 (0.006, 0.059)	0.081 (0.037, 0.120)	0.061
Kwon vs. ISAN	−0.018 (−0.052, 0.018)	−0.049 (−0.089, 0.010)	0.234
PANTHERIS vs. ISAN	−0.039 (−0.079, 0.001)	0.006 (−0.074, 0.087)	0.320
Chumbler vs. ISAN	0.007 (−0.048, 0.060)	0.020 (−0.014, 0.059)	0.696
Kwon vs. AIS-APS	−0.081 (−0.110, −0.053)	−0.058 (−0.014, 0.021)	0.568
PANTHERIS vs. AIS-APS	−0.075 (−0.11, −0.036)	−0.05 (−0.012, 0.024)	0.545
PANTHERIS vs. Kwon	−0.012 (−0.069, 0.052)	−0.000084 (−0.047, 0.046)	0.756
Chumbler vs. PANTHERIS	0.100 (0.037, 0.170)	0.029 (−0.015, 0.072)	0.065

### Network meta-analysis results

**Figure 6:**
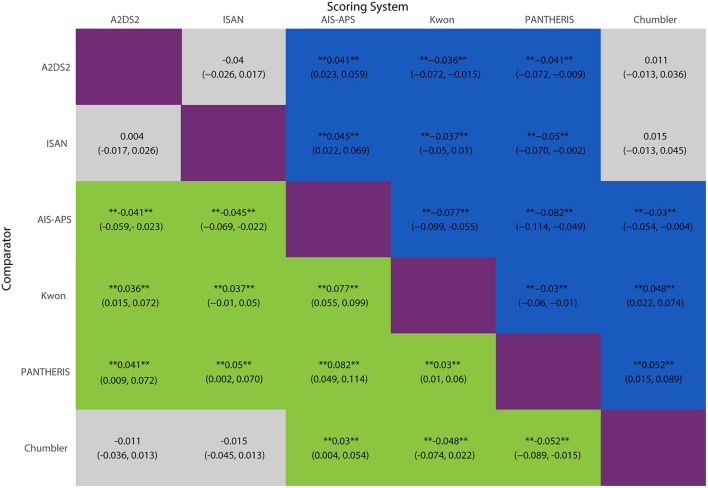
MD with 95% CIs of AUC of six risk scores for SAP. The values in each cell represent the relative effect of the risk score on the top, compared to the risk score on the left. **Indicates statistical significance.

The network meta-analysis results were shown in Figure 6. AIS-APS had excellent performance in prediction accuracy for SAP than Chumbler (MD = 0.030, 95%CI: 0.004, 0.054), A2DS2 (MD = 0.041, 95% CI: 0.023, 0.059), ISAN (MD = 0.045, 95% CI: 0.022, 0.069), Kwon (MD = 0.077, 95% CI: 0.055, 0.099) and PANTHERIS (MD = 0.082, 95% CI: 0.049, 0.114).

However, the results showed no significant differences between Chumbler, A2DS2 and ISAN (Chumbler: MD = 0.015, 95%CI: −0.013, 0.045, A2DS2: MD = 0.004, 95% CI: −0.017, 0.026, compared with ISAN), no significant differences between Kwon and PANTHERIS (Kwon: MD = 0.005, 95% CI: −0.031, 0.039, compared with PANTHERIS).

**Figure 7:**
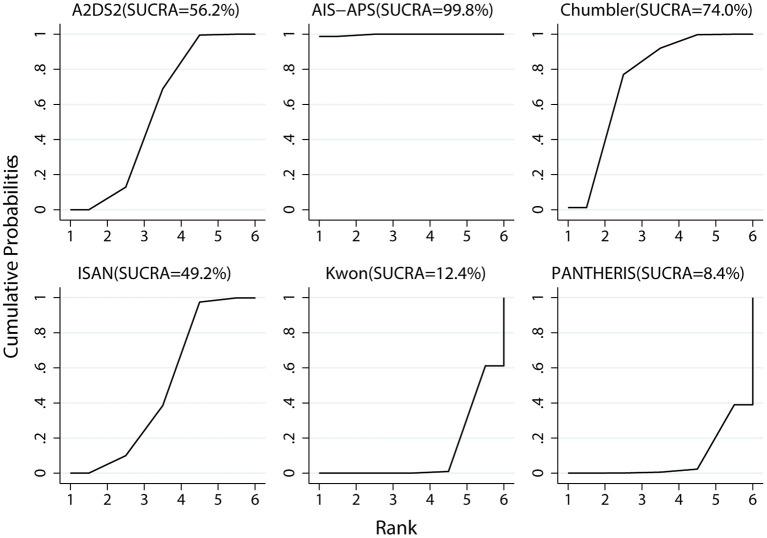
Plot of SUCRA.

Prediction accuracy ranking based on SUCRA values, were as follows: AIS-APS, Chumbler, A2DS2, ISAN, KWON, PANTHERIS. The details are depicted in Figure 7.

### Funnel plot characteristics

**Figure 8:**
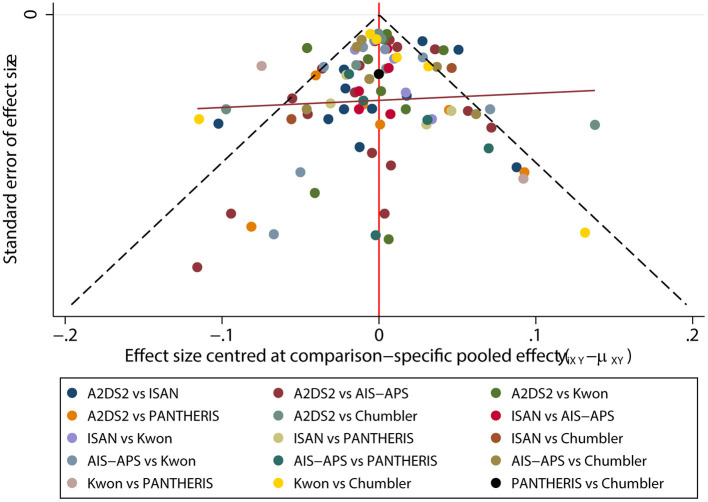
Funnel plot.

The funnel plot result was displayed in Figure 8. There were roughly symmetrical in visual, but some points lay outside the funnel, which revealed there was a small sample size and publication bias.

## Discussion

As the increasing morbidity and mortality of SAP, it is necessary to predict the SAP risk at the early stage. However, which scoring system of prediction is better has long been matter of debate. To the best of our knowledge, this is the first and the most comprehensive Bayesian NMA evaluating the scores for predicting SAP. The most important finding of our study is that the AIS-APS score showed better value in predicting the risk of SAP.

The AIS-APS score was derived by Ji Ruijun et al. in 2013, based on the China National Stroke Registry (CNSR), a nationwide, multicenter, and prospective registry of consecutive patients with acute cerebrovascular events. The following items were selected: age, past medical history, current smoking, modified Rankin Scale (mRS), Glasgow Coma Scale (GCS), National Institutes of Health Stroke Scale (NIHSS), Oxfordshire Community Stroke Project (OCSP), dysphagia, and admission glucose, with 5 risk stratifications [very low (0–6), low (7–13), intermediate (14–20), high (21–27), and very high (28–35)]. In the previous cohort (17), the AIS-APS had the highest associated sensitivity, specificity, positive predictive value and negative predictive value in the internal and external validation. The results of our study corroborate growing recent evidence that suggest the AIS-APS score had better predictive accuracies for SAP in comparative diagnostic accuracy studies. Zhang et al. (43) found that the AIS-APS [AUC: 0.79 (95% CI: 0.78–0.80)] performed superior to the A2DS2 score [AUC: 0.74 (95% CI: 0.73–0.75)] and the ISAN score [AUC: 0.76 (95% CI: 0.75–0.78)] to in predicting in-hospital pneumonia after AIS. Siregar et al. (46) found the AIS-APS [AUC: 0.792 (95% CI: 0.761–0.823)] performed a higher AUC value than the A2DS2 score for the Indonesia patients. The reliability and structural validity of the AIS-APS score were validated in the study of Qinxia et al. (49). The result of internal consistency (Cronbach's α) was 0.831. The results of the structural validity test were consistent with the ideal structure of the score. The Chinese consensus statement suggests using AIS-APS as a prediction tool for SAP. With several risk stratification, most reliable and accurate scoring system, AIS-APS would be greatly beneficial to patients and clinicians. However, there were little external validations of AIS-APS for non-Asian populations. The AIS-APS should be externally validated in geographically distinct population.

Chumbler et al. developed a 3-category scoring system (Chumbler score) to better identify patients at high risk of SAP, from a secondary analysis of a retrospective cohort study based on the Veterans Health Administration (VHA), which included the medical history of pneumonia, dysphagia, increasing NIHSS score, being found down at symptom onset, and age >70 years. The main difference among Chumbler's, AIS-APS and A2DS2 is the weighted value of NIHSS score, which are 14/19, 5/9 and 8/35, respectively. A meta-analysis had shown that patients with an NIHSS score >15 points had a 14.63-fold increased risk for pulmonary infection compared with patients whose NIHSS scores were <15 points (50). Besides, Chumbler's score has a unique valuable “being found down at symptomonset.” Falling down after stroke may lead to bone fracture and then resting in bed for long time which would increase the risk of SAP. However, the Chumbler score was from a secondary analysis of a retrospective cohort study, the score needs more validations.

The A2DS2 score was derived by Hoffmann et al in 2012. The score was performed in a large derivation and validation sample based on the Berlin Stroke Register (BSR) data. It did not include meaningful risk categories. The risk score showed good discrimination properties. Although the A2DS2 score had been externally validated in Asian and non-Asian populations, it was not always performing better than other scores. Ye et al. (34) found that A2DS2 (AUC: 0.776 (95% CI: 0.694–0.859)] performed inferior to AIS-APS (AUC: 0.829 (95% CI: 0.769–0.889)] and PANYHERIS [AUC: 0.818 (95% CI:0.750–0.885)]. Helmy et al. (13) reported that the A2DS2 score showed the highest AUC [0.85 (95% CI: 0.74–0.92)] compared with AIS-APS [0.798 (95% CI: 0.685–0.884)] and PANTHERIS [0.715 (95% CI: 0.595–0.817)] in Egypt populations. The A2DS2 score should be externally validated.

To simplify the scoring system, Harms et al. developed the PANTHERIS score based on the Berlin Neurological Intensive Care Unit. The leukocyte count was selected as one of the predictors. However, it was limited by the small sample size and all patients with middle cerebral artery infarction. This score did not include medical history and stroke patterns. The PANTERIS score was originally developed for patients with severe strokes admitted to neurocritical care units. Current published studies almost were designed for emergency department or neurology department patients. Further comparative diagnostic accuracy studies for different stroke severity patients are required.

Overall, all scores were derived from a retrospective analysis of registry-based studies. Two scores (AIS-APS and PANTHERIS) required laboratory variables. Two scores (Kwon score and PANTHERIS) were derived from relatively small single-center studies limiting their applicability. Two scores (AIS-APS and ISAN) selected pre-stroke neurological status as one of the predictors. Several scores (Chumbler, AIS-APS and ISAN) provided risk stratification. All of the scores did not include meaningful predictions of outcomes. The details of the six scoring systems were shown in Table 3. Most of the scores can be directly performed after admission or soon after admission. The PANTHERIS score needs more time to be evaluated because of the inclusion of systolic blood pressure within 24 h after admission. Dysphagia almost was preliminary evaluated soon after admission with water swallow test. If patients already suffered SAP according to the diagnosis criteria of guideline, risk scores could help us to prove the effect of these scores. The addition of items such as WBC and systolic blood pressure within 24 h after admission could improve the predictive performance, but have a chance of delay for predict SAP. However, beside PANTHERIS score, other scores could be evaluated at similar time after admission. The balance between better accuracy, additive items and time warrants further consideration.

**Table 3:** The details of the six scoring systems.

		**A2DS2**	**ISAN**	**AIS-APS**	**Kwon**	**PANTHERIS**	**Chumbler**
	Derivation cohort	**BSR**	**SSNAP**	**CNSR**	**Seoul**	**Berlin NICU**	**VHA**
Items	Size (*n*)	15,335	11,551	8,820	286	223	925
	Sex (Male)	+1	+1		+1		
	Age (year)	+1 (≥75)	+3 (60–69)	+2 (60~69)	+1 (≥65)	+1 (60–80)	+2 (>70)
			+4 (70–79)	+5 (70~79)		+2 (>80)	
			+6 (80–89)	+7 (≥80)			
			+8 (≥90)				
	Mechanical ventilation				+1		
	Atrial fibrillation	+1		+1			
	Congestive heart failure			+3			
	COPD			+3			
	Current smoking			+1			
	Dysphagia	+2		+3	+1		+4
	Past medical history of pneumonia						+4
	NIHSS	+3 (5–15)	+4 (5–15)	+2 (5–9)	+1 (≥11)		+1 (per 3 increase)
		+5 (≥16)	+8 (16–20)	+5 (9–14)			
			+10 (≥21)	+8 (≥15)			
	Found down at symptom onset						+3
	mRS (perstroke)		+2 (2–5)	+2 (≥3)			
	GCS			+3 (3~8)		+2 (9–12)	
						+5 (3–8)	
	WBC ( × 109/L)					+3 (>11)	
	Systolic blood pressure (within 24 h after admission)					+2 (>200 mmHg)	
	OCSP (TACI/POCI)			+2			
	Admission glucose (mmol/L)			+2 (≥11.1)			
	Total score	0–10	0–21	0–35	0–5	0–12	0–27

BSR, Berlin Stroke Register; SSNAP, Sentinel Stroke National Audit Programme; CNSR, China National Stroke Registry; NICU, Neurological Intensive Care Unit; VHA, Veterans Health Administration; COPD, indicates chronic obstructive pulmonary disease; GCS, Glasgow Coma Scale; mRS, modified Rankin Scale; NIHSS, National Institutes of Health Stroke Scale; OCSP, Oxfordshire Community Stroke Project; TACI, Total anterior circulation infarct; POCI, Posterior circulation infarct.

Initially, we wanted to analyze the sensitivity and specificity of each score. However, most of the studies did not provide 95%CIs and the standard deviation of sensitivity and specificity. Meanwhile, true positive rate, false positive rate, false negative rate, and true negative rate were also missing in several studies. After trying to contact with authors, we still did not have enough data available for the analysis. And the network meta-analysis methodology of diagnostic accuracy studies was still inadequate. We finally gave up on the analysis.

Preventive antibiotics had not shown any effect neither in reducing the incidence of stroke-associated pneumonia nor decreasing the mortality or improving the proportion of good outcomes within this field for the last decade. A systematic review (51) made an analysis of preventive antibiotics in patients with different risk score (A2DS2 or ISAN) and found risk scores did not significantly influence treatment response of preventive antibiotic therapy. However, few clinical trials have compared patients with different risk scores. None of the studies investigated the analysis of the impact of risk scores on clinical outcomes. We expected more future clinical trials could include risk scores as an inclusion criterion and prove the effect of preventive antibiotics in patients with stroke. In addition, except preventive antibiotics, lots of other intervention could prevent SAP in patients with stroke of high risk. For instance, dysphagia assessment and management (52), modes of nutritional support (53, 54) and care programme (55) also play important parts in the prevention of SAP.

## Limitation

This study also has some limitations. First, most of the studies are based on Chinese patients, which may lead to publication bias and affect the validity and reliability of this systematic review. Second, the number of studies for some scoring systems are low, which may affect the comparison with others.

## Conclusion

In conclusion, the AIS-APS is a validated clinical tool for predicting SAP after the onset of acute ischemic stroke. Due to the limitations of this study, the results should be verified by more multi-center and large-sample prospective studies and geographically distinct populations.

## Data availability statement

The original contributions presented in the study are included in the article/Supplementary material, further inquiries can be directed to the corresponding author/s.

## Author contributions

XZ: conceptualization, methodology, software, formal analysis, quality assessment, investigation, resources, data curation, writing—original draft preparation, review and editing, and visualization. LX: conceptualization, methodology, validation, formal analysis, quality assessment, investigation, data curation, writing—original review and editing, and visualization. LN: investigation, resources, data curation, and software. YT: methodology, validation, data curation, quality assessment, writing—original review and editing, and supervision. KC: writing—original review and editing, supervision, and project administration. All data were generated in-house and no paper mill was used. All authors listed have made a substantial, direct, and intellectual contribution to the work and approved it for publication.

## Conflict of interest

The authors declare that the research was conducted in the absence of any commercial or financial relationships that could be construed as a potential conflict of interest.

## Publisher's note

All claims expressed in this article are solely those of the authors and do not necessarily represent those of their affiliated organizations, or those of the publisher, the editors and the reviewers. Any product that may be evaluated in this article, or claim that may be made by its manufacturer, is not guaranteed or endorsed by the publisher.

## Supplementary material

Attachment 1 PRISMA NMA checklist.

Attachment 2 Search strategy.

## Abbreviations

A2DS2, Age, Atrial fibrillation, Dysphagia, Sex: Stroke Severity
ISAN, Prestroke Independence, Sex, Age: National Institutes of Health Stroke Scale
AIS-APS: Acute Ischaemic Stroke-Associated Pneumonia Score
PANTHERIS: Preventive Antibacterial Therapy in Acute Ischaemic Stroke
AUC: Area under the receiver operating characteristic
BSR: Berlin Stroke Register
CI: Confidence interval
CNSR: China National Stroke Registry
COPD: indicates chronic obstructive pulmonary disease
DIC: Deviance information criterion
GCS: Glasgow Coma Scale
MCMC: Markov chain Monte Carlo
MD: Mean differences
mRS: modified Rankin Scale
NICU: Neurological Intensive Care Unit
NIHSS: National Institutes of Health Stroke Scale
NMA: Network meta-analysis
OCSP: Oxfordshire Community Stroke Project
OR: Odds ratios
POCI: Posterior circulation infarct
PRISMA: Preferred Reporting Items for Systematic reviews and Meta-Analyses
PSRF: Potential scale reduction factor
SAP: Stroke-associated pneumonia
SUCRA: Surface under the cumulative ranking curves
SSNAP: Sentinel Stroke National Audit Programme
TACI: Total anterior circulation infarct
TIA: Transient ischemic attack
UME: Unrelated mean effects
VHA: Veterans Health Administration.
