# Supplementary material for: Comparison of six risk scores for stroke-associated pneumonia in patients with acute ischemic stroke: A systematic review and Bayesian network meta-analysis
Source: Front Med (Lausanne). 2022 Oct 12;9:964616. doi: 10.3389/fmed.2022.964616 (PMC9596973; doi:10.3389/fmed.2022.964616)
Supplement: Attachment 2 — Search strategy. [file Data_Sheet_2.docx]

**Attachment 2: Search Strategy**

PubMed

| Number | Search terms | Results |
| --- | --- | --- |
| #1 | A2DS2[All Fields] | 22 |
| #2 | ISAN[All Fields] | 363 |
| #3 | AIS-APS[All Fields] | 8 |
| #4 | Kwon[All Fields] | 44313 |
| #5 | Pneumonia score[All Fields] | 18544 |
| #6 | PANTHERIS[All Fields] | 24 |
| #7 | Chumbler[All Fields] | 100 |
| #8 | VHA score[All Fields] | 255 |
| #9 | #1 OR #2 OR #3 OR #4 OR #5 OR #6 OR #7 OR #8 | 63509 |
| #10 | Pneumonia [All Fields] | 386125 |
| #11 | Lung Inflammation [All Fields] | 379075 |
| #12 | lower respiratory infection [All Fields] | 50635 |
| #13 | lower respiratory tract infection [All Fields] | 45356 |
| #14 | low respiratory tract infection [All Fields] | 35806 |
| #15 | lower airway infection [All Fields] | 5663 |
| #16 | #10 OR #11 OR #12 OR #13 OR #14 OR #15 | 479183 |
| #17 | stroke[All Fields] | 398010 |
| #18 | #9 AND #16 AND #17 | 826 |

Web of Science

| Number | Search terms | Results |
| --- | --- | --- |
| #1 | ALL:A2DS2 | 9 |
| #2 | ALL:ISAN | 1249 |
| #3 | ALL:AIS-APS | 8 |
| #4 | ALL:Kwon | 91874 |
| #5 | ALL: Pneumonia score | 10374 |
| #6 | ALL:PANTHERIS | 25 |
| #7 | ALL:Chumbler | 81 |
| #8 | ALL:VHA score | 295 |
| #9 | #1 OR #2 OR #3 OR #4 OR #5 OR #6 OR #7 OR #8 | 103819 |
| #10 | ALL: Pneumonia | 119126 |
| #11 | ALL: Lung Inflammation | 94965 |
| #12 | ALL: lower respiratory infection | 34131 |
| #13 | ALL: lower respiratory tract infection | 14239 |
| #14 | ALL: low respiratory tract infection | 14146 |
| #15 | ALL: lower airway infection | 4870 |
| #16 | #10 OR #11 OR #12 OR #13 OR #14 OR #15 | 235893 |
| #17 | ALL:stroke | 539472 |
| #18 | #9 AND #16 AND #17 | 769 |

Embase

| Number | Search term | Results |
| --- | --- | --- |
| #1 | A2DS2:ti,ab,kw OR ISAN:ti,ab,kw OR ‘AIS-APS’:ti,ab,kw OR Kwon:ti,ab,kw OR ‘Pneumonia score’:ti,ab,kw OR PANTHERIS:ti,ab,kw OR Chumbler:ti,ab,kw OR ‘VHA score’ | 992 |
| #2 | pneumonia:ti,ab,kw OR 'lung inflammation':ti,ab,kw OR 'lower respiratory infection':ti,ab,kw OR 'lower respiratory tract infection':ti,ab,kw OR 'low respiratory tract infection':ti,ab,kw OR 'lower airway infection':ti,ab,kw | 225220 |
| #3 | stroke:ti,ab,kw | 453921 |
| #4 | #1 AND #2 AND #3 | 48 |

CNKI

| Number | Search term | Results |
| --- | --- | --- |
| #1 | SU='A2DS2' OR SU='ISAN' OR SU='AIS-APS' OR SU='Kwon' OR SU=’ Pneumonia score’ OR SU='Chumbler' OR SU=’ VHA score’ OR SU='PANTHERIS' | 289 |
| #2 | SU=' Pneumonia ' OR SU=' Lung Inflammation ' OR SU='ower respiratory infection' | 264157 |
| #3 | SU=' stroke ' OR SU=' cerebral infarction ' OR SU=' infarction of brain ' OR SU= “cerebrovascular disease“ OR SU=' cerebrovascular accident ' | 306577 |
| #4 | #1 AND #2 AND #3 | 40 |

Wanfang Data

| Number | Search term | Results |
| --- | --- | --- |
| #1 | TOPIC:("A2DS2" OR "ISAN" OR "AIS-APS" OR "Kwon" OR "Pneumonia score" OR "Chumbler" OR "VHA score" OR "PANRTHERIS") | 2666 |
| #2 | TOPIC: ("Pneumonia " OR " Lung Inflammation " OR " lower respiratory infection ") | 478352 |
| #3 | TOPIC: ("stroke " OR " infarction of brain " OR "cerebral infarction" OR " cerebrovascular disease " OR " cerebrovascular accident ") | 650176 |
| #4 | #1 AND #2 AND #3 | 70 |

VIP

| Number | Search term | Results |
| --- | --- | --- |
| #1 | U=(A2DS2 OR ISAN OR "AIS-APS" OR Kwon OR "Pneumonia score" OR Chumbler OR "VHA score"OR PANTHERIS) | 2948 |
| #2 | U=(Pneumonia OR “Lung Inflammation” OR “lower respiratory infection”) | 362193 |
| #3 | U=(stroke OR “cerebral infarction” OR “infarction of brain” OR “cerebrovascular disease” OR “cerebrovascular accident”) | 305201 |
| #4 | #1 AND #2 AND #3 | 254 |

Sinomed(CBM)

| Number | Search terms | Results |
| --- | --- | --- |
| #1 | ("A2DS2"[All Fields] OR "ISAN"[All Fields] OR "AIS-APS"[All Fields] OR "Kwon"[All Fields] OR "Pneumonia score"[All Fields] OR "PANTHERIS"[All Fields] OR "Chumbler "[All Fields] OR "VHA score"[All Fields]) | 345 |
| #2 | (" Pneumonia "[All Fields] OR " Lung Inflammation "[All Fields] OR " lower respiratory infection "[ All Fields]) | 241835 |
| #3 | (" stroke "[All Fields] OR "cerebral infarction"[All Fields] OR " infarction of brain "[All Fields] OR "cerebrovascular disease "[All Fields] OR " cerebrovascular accident "[All Fields]) | 341109 |
| #4 | #1 AND #2 AND #3 | 33 |
